# Effect of whole-body vibration stimulation on plasma soluble TNF receptors in elderly with sarcopenia: a randomized controlled trial

**DOI:** 10.1590/1414-431X2024e13282

**Published:** 2024-04-19

**Authors:** H.C. Almeida, V.K.S. Lage, R. Taiar, J.M. Santos, F.A. de Paula, A. Rapin, D.C. Sá-Caputo, M. Bernardo-Filho, A.C.R. Lacerda, V.A. Mendonça

**Affiliations:** 1 Programa de Pós-Graduação Multicêntrico em Ciências Fisiológicas, Sociedade Brasileira de Fisiologia, Universidade Federal dos Vales do Jequitinhonha e Mucuri, Diamantina, MG, Brasil; 2 Programa de Pós-graduação em Ciências da Saúde, Universidade Federal dos Vales do Jequitinhonha e Mucuri, Diamantina, MG, Brasil; 3 MATIM, Moulin de la Housse, Université de Reims Champagne Ardenne, Reims, France; 4 Centre Hospitalo-Universitaire de Reims (CHU Reims), Hôpital Sébastopol, Département de Médecine Physique et de Réadaptation, Reims, France; 5 Université de Reims Champagne-Ardenne, Faculté de Médecine, Reims, France; 6 Laboratório de Vibrações Mecânicas e Práticas Integrativas, Departamento de Biofísica e Biometria, Instituto de Biologia Roberto Alcântara Gomes e Policlínica Piquet Carneiro, Universidade do Estado do Rio de Janeiro, Rio de Janeiro, RJ, Brasil

**Keywords:** Whole-body vibration, Sarcopenia, Inflammation, Elderly, TNFR

## Abstract

Sarcopenia is a pathology resulting from a progressive and severe loss of muscle mass, strength, and function in the course of aging, which has deleterious consequences on quality of life. Among the most widespread studies on the issue are those focused on the effect of different types of physical exercise on patients with sarcopenia. This randomized controlled study aimed to compare the effects of a whole-body vibration exercise (WBV) session on the inflammatory parameters of non-sarcopenic (NSG, n=22) and sarcopenic elderly (SG, n=22). NSG and SG participants were randomly divided into two protocols: intervention (squat with WBV) and control (squat without WBV). After a one-week washout period, participants switched protocols, so that everyone performed both protocols. Body composition was assessed by dual-energy radiological absorptiometry (DXA) and function through the six-minute walk test (6MWD) and Short Physical Performance Battery (SPPB). Plasma soluble tumor necrosis factor receptors (sTNFR) were determined by enzyme-linked immunosorbent assay (ELISA) and measured before and immediately after each protocol. After exercise with WBV, there was an increase in sTNFR2 levels in the NSG (P<0.01; d=-0.69 (-1.30; -0.08) and SG (P<0.01, d=-0.95 (-1.57; -0.32) groups. In conclusion, an acute session of WBV influenced sTNFr2 levels, with sarcopenic individuals showing a greater effect. This suggested that WBV had a more pronounced impact on sTNFr2 in those with loss of muscle strength and/or physical performance. Additionally, WBV is gaining recognition as an efficient strategy for those with persistent health issues.

## Introduction

Population aging is a global reality, and with it, chronic degenerative diseases are emerging as an increasingly common challenge for health professionals (1). Sarcopenia comes out in this scenario as a complex geriatric condition, currently recognized as a progressive and generalized muscle disease (CID-10 M62.5), which results in loss of muscle mass, strength, and function (2). Sarcopenia leads to reduced quality of life, functional decline, increased incidence of falls, poorer performance in activities of daily living (ADLs), and death (3). It causes important healthcare costs and is a public health problem with an undesirable economic impact (4).

The prevalence of sarcopenia is difficult to determine, varying according to the population and the evaluation criteria of the studies. A meta-analysis revealed a prevalence ranging from 9% in women to 11% in older men living in the community; 31% in women and 51% of men living in long-term care facilities for the elderly and 24% in women and 23% older men that are hospitalized (5).

The pathophysiology of sarcopenia is complex and multifactorial, involving muscle changes that lead to the accumulation of pro-inflammatory cytokines and reactive oxygen species (ROS), hormonal disorders, defective autophagy, and dysfunction of muscle satellite cells. In addition, external factors such as sedentary lifestyle and malnutrition (5) are involved, promoting imbalance between muscle protein synthesis and degradation, with prevalence of the latter (6).

Sarcopenia affects the immune response, leading to the development of chronic low-grade inflammation (LGI), which results in the constant release of pro-inflammatory cytokines, among which tumor necrosis factor (TNF) stands out (7). In this context, LGI due to aging is known as “inflammaging”, also with TNF as one of the main cytokines involved (8). Evidence suggests that TNF and its soluble receptors (sTNFr1 and sTNFr2) participate in the regulation of muscle mass, accelerating protein degradation and leading to muscle weakness (9). While sTNFr1 is linked to pro-inflammatory and apoptotic effects, sTNFr2 has been linked to a variety of immunoregulatory and anti-inflammatory functions. Importantly, a complex interaction between sTNFr1 and sTNFr2 has been described and additive, synergistic, and antagonistic effects have been demonstrated (10,11).

Evidence suggests that sarcopenia is a reversible cause of disability and may benefit from interventions, especially in its early stages. Considering the deleterious consequences of sarcopenia on the quality of life and functionality of individuals, it is essential to establish effective and viable forms of prevention and treatment. Among the most widespread and validated by scientific studies are nutrition and physical exercise (11).

The squat is an effective exercise to prevent sarcopenia by improving functional performance and facilitating postural stabilization of the trunk (12). Studies have shown that squat resistance training was effective in increasing muscle mass and strength in older adults (12-14).

Although resistance training is a primary intervention to decrease the effects of sarcopenia (15,16), whole-body vibration exercise (WBV) has gained considerable attention in recent years as a safe, adequate, and effective method for the older population (17). Several beneficial effects have been associated with WBV exercise and neuromuscular responses have been described, such as an increase in muscle strength, power, and flexibility, improvement in balance, cardiorespiratory fitness, inflammatory profile, oxidative stress, bone mineral density, and a decrease in the risk of falls (16,18-22).

Many studies applied the WBV stimulus associated with the squat, since this association seems to intensify the neuromuscular responses through the activation of the tonic vibrating reflex, increasing the synchrony of the motor units and providing greater neuromuscular efficiency (23,24).

The adaptations of the different physiological systems and the inflammatory response to physical training result from the sum of the changes triggered by each exercise session, which makes it important to understand the acute changes of a single WBV session. The objective of this study was to compare the effect of a single WBV session on inflammatory parameters in sarcopenic and non-sarcopenic elderly people.

## Material and Methods

### Participants and sample size estimation

A crossover randomized controlled trial was conducted between February 2018 and January 2020, with older people from the general community of the city of Diamantina, MG, Brazil Participants were recruited through verbal invitation, leaflets, and visits to primary healthcare units and physicians' offices, or through communication (internet, radio). The inclusion criteria were people aged 60 years or over of either sex, who met the criteria of sarcopenia diagnosis, according to the cutoff points of relative skeletal muscle index (RSMI) described by the European Working Group Consensus on Sarcopenia in Older People - EWGSOP (2). Exclusion criteria were: 1) acute illness; 2) decompensated chronic disease; 3) taking immunosuppressive and/or anti-inflammatory and/or beta-blocker medication; 4) participation in any physical training program three months prior the beginning of the assessments; 5) contraindication to the vibrating platform, such as epilepsy, gallstones or kidney stones, neuromuscular and neurodegenerative diseases, stroke, serious heart disease, or those with an implant, bypass, or stent; and 6) cognitive impairment. This study followed the recommendations proposed by the CONSORT statement (25) and was approved by the Ethics Committee of the Universidade Federal dos Vales do Jequitinhonha e Mucuri, Identification No. 74422817.1.0000.5108 and registered with the Brazilian Clinical Trials Registry (REBEC; RBR-35w9bw). Informed consent was obtained from all the participants.

The sample size was determined according to the study of Ribeiro et al. (20). Considering an effect size of P=0.05, Cohens-d=0.47, and power of 0.94, found by two-way ANOVA, the estimated sample size was 18 individuals per group (non-sarcopenic and sarcopenic). The value obtained was increased by 11% to compensate for potential losses during the execution of the project, with 22 individuals per group, totaling 44 participants. The distribution of participants between the groups was controlled by sex, age, and drug class, to minimize the influence of confounding variables.

**Figure 1:**
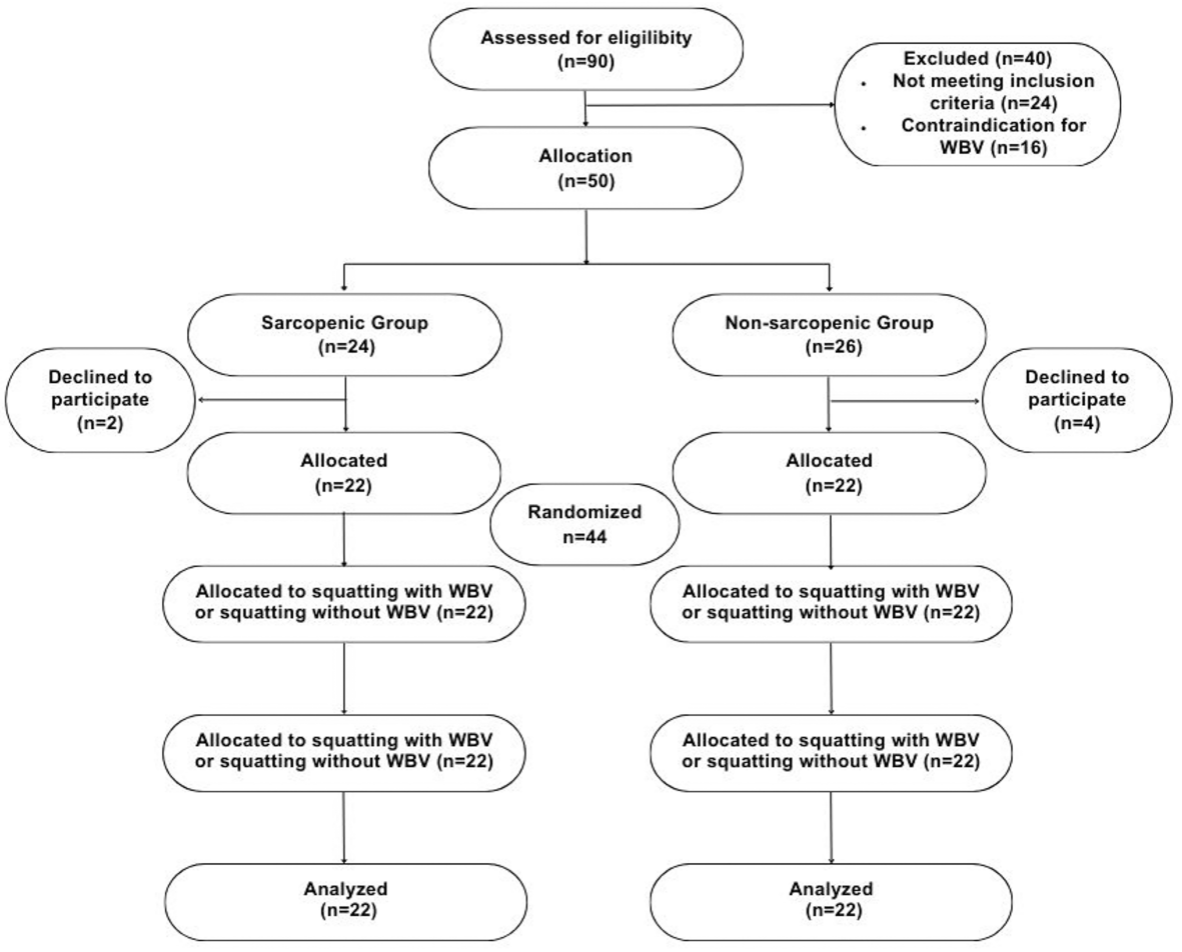
Flowchart of study participants. WBV: whole-body vibration exercise.

All the 44 participants completed the study and were included in the analysis as allocated (intention-to-treat analysis). Each participant went through both protocols, in a randomized and cross-over design (Figure 1).

### Diagnostic criteria for sarcopenia

Participants were evaluated using dual-energy X-ray absorptiometry (DXA) (Lunar, DPX, USA) for body composition measurements, and for the diagnosis of sarcopenia, relative skeletal muscle index (RSMI) cutoff points were considered using appendicular skeletal muscle mass (ASM) divided by height squared. The cutoff point for the diagnosis of sarcopenia was <7.0 kg/m^2^ for men and <5.5 kg/m^2^ for women (2,26).

### Procedures

All experimental procedures were performed in the Universidade Federal dos Vales do Jequitinhonha e Mucuri (UFVJM, Brazil) and conducted by trained and qualified researchers.

#### Body mass index (BMI)

Body mass and height were measured using a scale with stadiometer. BMI was calculated by dividing body mass (kg) by the square of height (m), adopting the cutoff point for eutrophic BMI between 22 and 27 kg/m^2^ (27). Waist circumference also was measured based on the I Diretriz Brasileira de Diagnóstico e Tratamento da Síndrome Metabólica (First Brazilian Guideline for Diagnosis and Treatment of Metabolic Syndrome) (28).

#### Body composition assessment

Total body mass, fat mass, lean mass, ASM, and free fat mass (FFM) were assessed using DXA. Fat mass and lean mass were assessed through total body analysis and by body segment (upper, lower, and arms and legs).

#### Functionality assessment

To assess functionality, the following tests were performed: Short Physical Performance Battery (SPPB), which is a functional performance test composed of standing static and balancing in three positions (side-by-side stands, semi-tandem, and tandem), 4-m gait speed test (where the cutoff point used was 0.8 m/s (2) and sit-to-stand (5STS) test, which measures the time taken to sit and and stand from a chair without using the arms five times (29). The cutoff point for SPPB is 8 (2) for a diagnosis suggestive of functional impairment. In addition, handgrip strength (HS) was measured using the Jamar^®^ (USA) dynamometer, with cutoff points for sarcopenia for low handgrip strength being <27 kg and <16 kg for men and women, respectively (2). Also, the 6-min walking distance (6MWD) was measured (walk as fast as possible during six minutes) (30). The assessors were blinded in relation to the groups in which the participants were included.

### Interventions

All the experimental procedures were conducted in the same place and on a set schedule. Participants were stratified according to the diagnosis of sarcopenia into the non-sarcopenic group (NSG) and the sarcopenic group (SG). The order of execution of the two experimental situations was randomized by the researchers through a simple draw, in which the protocols were marked by numbers, whereby number 1 corresponded to squatting with WBV and number 2 to squatting without WBV. The participants were randomly allocated to one of the protocols, and after a washout period of one week, they performed the other intervention. All participants performed the intervention protocol (squat with WBV stimulus) and the control protocol (squat without WBV).

#### Exercise intervention with WBV

The vibration exposure consisted of performing dynamic squatting exercises (8 sets of 40 s) with a mechanical vibration stimulus (frequency of 40 Hz and amplitude of 4 mm) performed on a commercial model of a vibration platform (VP) (FitVibe^®^, GymnaUniphy NV, Belgium) (23). The mechanical vibration frequency and amplitude were selected due to prototype renders of an acceleration range of 2-5 *g* (31). The participants were instructed to perform 3 s of isometric flexion at a 60° angle and 3 s of isometric flexion of the knees at a 10° angle. Between the sets, the participants rested for 40 s in the orthostatic position on the turned-off VP. The 60° angle was measured for each volunteer using a universal goniometer before initiating the exercise sets. A barrier was placed at the gluteal region to limit the flexion degree of the knees (20). The exercise execution time was around 10 min.

#### Exercise intervention without WBV

The without-WBV intervention group performed the same dynamic squatting exercises (8 sets of 40 s) following the same procedures as described for the intervention with WBV but with the VP turned off.

### Data collection

#### Inflammatory measurements

Blood samples were collected at rest after fasting. A standardized breakfast was offered, and participants performed the interventions after eating. Another blood sample was collected immediately after the interventions. Blood was collected aseptically in tubes containing EDTA anticoagulants (10 mL of blood). The tubes were centrifuged at 1008 *g* at 4°C for 10 min. Plasma samples were stored in a freezer at -80°C until the moment of the experiments. Plasma soluble TNF receptors (sTNFR1 and sTNFR2) were measured using conventional sandwich ELISA kits (DuoSet, R&D Systems, USA), according to the manufacturer's instructions. Detection limits were 5.0 pg/mL for kits.

### Statistical analysis

Statistical analysis was performed by STATA 15 (Data Analysis and Statistical Software College Station, USA) and R Core Team software (USA). The normality and homogeneity of variances were verified using Kolmogorov-Smirnov and Levene's tests.

To compare the differences between the non-sarcopenic and sarcopenic groups for anthropometric variables and functional tests, the independent *t*-test was used, followed by the differences between the means for the two groups. The size of the difference was calculated by the magnitude of the effect (Cohen's d). Differences in soluble TNF receptors were compared before and after exercise, with and without WBV, for the non-sarcopenic and sarcopenic groups using paired *t*-test, followed by the delta of the differences between the means of after and before exercises and measures of the magnitude of the effect of this difference. An effect size of 0.8 and P<0.05 were considered statistically significant. Data are reported as means±SE.

## Results

The 44 participants included in this study were divided into NSG (n=22) and SG (n=22) groups for analysis (Figure 1). The mean age was 72.0±7.2 and 71.6±7.9 years in the NSG and SG groups, respectively, and each group had participants of both sexes (11 men and 11 women). The anthropometric and body composition values were lower in SG compared to NSG (P<0.05), except for values of age and body fat. The characteristics of the participants of NSG and SG groups are shown in Table 1.

**Table 1:** Comparison of demographic, anthropometric, and physical test variables between non-sarcopenic (NSG) and sarcopenic (SG) groups.

Variables	NSG	SG	P-value	Cohen's d	95%CI
Demographic and anthropometric					
Age (years)	72.0±7.2	71.6±7.9	0.86	0.05	-0.53; 0.64
Weight (kg)	60.9±8.6	51.3±8.0	<0.01*	1.14	0.49; 1.77
BMI (kg/m^2^)	24.9±2.5	21.3±2.0	<0.01*	1.53	0.85; 2.20
WC (cm)	89.0±6.5	81.1±9.0	<0.01*	1.01	0.37; 1.63
Body fat (%)	32.2±6.4	30.9±8.7	0.59	0.16	-0.43; 0.75
Fat mass (kg)	18.8±3.9	15.2±4.7	0.01*	0.83	0.21; 1.45
Lean mass (kg)	40.0±7.3	33.9±6.8	0.01*	0.86	0.24; 1.47
FFM (kg)	41.1±7.4	34.9±6.9	0.01*	0.86	0.23; 1.47
RSMI (kg/m^2^)	7.1±1.0	5.8±0.7	<0.01*	1.44	0.77; 2.10
RSMI, men	8.0±0.6	6.5±0.4	<0.001**	2.60	1.38; 3.77
RSMI, women	6.4±0.6	5.2±0.3	<0.001**	2.51	1.35; 3.63
Physical tests					
6MWD (m)	524.0±70.0	469.6±78.3	0.02*	0.73	0.11; 1.33
SPPB (score)	10.8±1.00	10.1±1.47	0.06	0.57	0.03; 1.17
5STS (s)	9.01±1.61	10.85±2.51	0.01*	-0.87	-1.48; -0.24

Data are reported as means±SE. P*-*values derived from independent *t*-tests. *P<0.05 or **P<0.01. BMI: body mass index; WC: waist circumference; FFM: free fat mass; RSMI: relative skeletal muscle index; 6MWD: 6-min walking distance test; SPPB: Short Physical Performance Battery; 5STS: five times sit-to-stand test.

**Figure 2:**
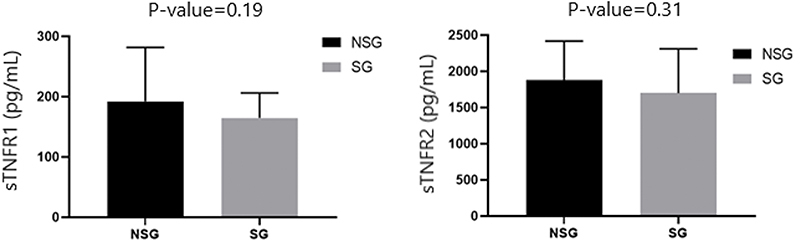
Comparison of soluble tumor necrosis factors (sTNF) 1 and 2 receptors between non-sarcopenic (NSG) and sarcopenic (SG) groups. Data are reported as means±SE. P-values are derived from the independent *t*-test.

The SG presented poorer performance in 6MWD and 5STS compared to NSG (Table 1). Furthermore, plasma levels of sTNFR1 and sTNFR2 were similar in the NSG and SG (P=0.05) (Figure 2).

Table 2 shows the comparison of biomarkers before and after exercise without and with WBV. In NSG, no differences were found between before and after exercise without WBV for sTNFR1 and sTNFR2 (P>0.05). However, SG had an increase in sTNFR2 levels after exercise without WBV [P=0.03, d=-0.48 (-1.08; 0.11)]. After the exercise with WBV, an increase in sTNFR2 levels was found in NSG [P<0.01; d=-0.69 (-1.30; -0.08)] and in SG [P<0.01, d=-0.95 (-1.57; -0.32)].

**Table 2:** Comparison of biomarkers before and after exercise without WBV and with WBV in non-sarcopenic and sarcopenic groups.

	Before	After	Δ	P-value	Cohen's d	95%CI
Without WBV						
Non-sarcopenic group						
sTNFr1 (pg/mL)	229.8±183.9	202.0±103.3	-27.8	0.54	0.18	-0.40; 0.77
sTNFr2 (pg/mL)	2195.0±1331.2	2175.1±585.9	-19.8	0.95	0.02	-0.57; 0.61
Sarcopenic group						
sTNFr1 (pg/mL)	210.3±71.9	211.6±68.8	+1.2	0.94	-0.01	-0.60; 0.57
sTNFr2 (pg/mL)	1958.5±683.6	2233.4±420.6	+274.8	0.03*	-0.48	-1.08; 0.11
With WBV						
Non-sarcopenic group						
sTNFr1 (pg/mL)	192.1±89.4	196.4±61.9	+4.29	0.62	-0.05	-0.64; 0.53
sTNFr2 (pg/mL)	1879.0±538.8	2254.1±538.6	+375.0	<0.01*	-0.692	-1.30; -0.08
Sarcopenic group						
sTNFr1 (pg/mL)	164.7±41.5	193.7±57.4	+29.0	0.06	-0.57	-1.18; 0.02
sTNFr2 (pg/mL)	1698.1±614.1	2204.4±427.0	+506.2	<0.01*	-0.95	-1.57; -0.32

Data are reported as means±SE. P*-*values for each group were derived from paired *t*-test. *P<0.05. WBV: Whole body vibration; sTNFR1: soluble tumor necrosis alpha receptor-1; sTNFR2: soluble tumor necrosis alpha receptor-2. Δ is the difference between after and before values.

## Discussion

Singh et al. (32) reported that the increase in plasma sTNFR1 levels is inversely proportional to the parameters of muscle mass, muscle strength, and physical function. However, this original work is the first study to analyze how a single WBV exercise session affects sTNFr1 and sTNFr2 parameters in older people divided into sarcopenic and non-sarcopenic groups.

SG had significantly lower anthropometric parameters and worse functional performance - indicated by the shorter distance covered in the 6MWT and the longer time in the 5STS test - compared to NSG. At baseline, NSG and SG had similar plasma sTNFr1 and sTNFr2 levels.

TNF-α has a unique feature compared to other cytokines, as it acts directly on skeletal muscle to decrease force. In addition to this specific action, TNF-α also stimulates the muscle to produce cytosolic oxidants at an accelerated rate (33). Muscle responses to TNF-α are triggered by activation of TNFR1 rather than TNFR2 (34). Evidence suggests that TNF-α and its sTNFr1 receptor are related to muscle weakness and have a negative impact on functional activities (9). These changes explain the worse results of body composition and functional capacity of individuals in the sarcopenic group found in the present study.

The plasma sTNFr1 levels did not change significantly with the exercise with or without WBV in the NSG. Plasma sTNFr1 levels increased only in the SG after WBV stimulation (P=0.06; Cohen-d=-0.57), which seems to point to a possible transient increase in sTNFr1 in response to the physical stimulus of exercise, which is important for triggering subsequent homeostatic responses.

The values of sTNFr2, on the other hand, increased significantly in the SG compared to baseline values after exercise without WBV, with a small effect size (P=0.03; Cohens-d=0.48). With WBV, the increase in sTNFr2 values compared to baseline was maintained (P<0.01; Cohens-d=0.95), but with a larger effect size. These results demonstrated efforts to achieve homeostasis, probably due to a transient inflammatory response. As there was an increase in sTNFr1 levels in the SG with combined WBV exercise, these results of a greater increase of sTNFr2 in SG, also in response to WBV, are consistent, since after acute exercises, there is an increase in the circulating levels of anti-inflammatories (IL-10, IL1-ra, and sTNFr2; 35).

Some studies demonstrate that physical exercise has an impact on inflammatory responses in the short and long term, depending on the type of exercise and its duration (36,37). The stimulus during and shortly after physical exercise can cause an increase in pro- and anti-inflammatory cytokines. However, regular exercise causes greater anti-inflammatory adaptations that are maintained in the long term, after the resolution of the acute inflammation (38). The literature has focused mainly on the analysis of these effects after resistance or cardio exercises, with scarce studies on WBV, which has been emerging as a safe and effective option for patients with chronic health conditions. When it comes to sarcopenia, the studies are even more limited.

Cristi et al. (37), in a study carried out with untrained elderly people, showed that there was no change in inflammatory markers (C-reactive protein, IL-6, IL-1, IL-10, and TNF-α) after 9 weeks of training with WBV. In an 8-week WBV program with elderly, Rodriguez-Miguelez et al. (39) found decreased TNF-α values. Decreased TNF-α values (50.9%) were also reported in the study by Oh et al. (40), who developed a program to perform WBV exercise for 6 months in patients with non-alcoholic fatty liver disease. In these studies, only TNF-α was measured and not its receptors.

Ribeiro et al. (20) reported a decrease in plasma sTNFR1 levels and an increase in sTNFR2 levels in individuals with fibromyalgia, while an increase in plasma levels of sTNFR1 was found in healthy women after only a single session of WBV exercise.

Lage et al. (22) investigated the acute effects of WBV stimulation in inflammatory markers including sTNF receptors in chronic obstructive pulmonary disease (COPD) patients. The authors found no differences in receptor concentration after WBV, in contrast to our results. Although both COPD and sarcopenia are chronic diseases that present low-grade inflammatory processes, it appears that the responses to WBV are different depending on the characteristics of the disease. The aforementioned study (22) found an increase in plasma concentrations of IL-10 after WBV, while in the current study, there was an increase in sTNFR2 levels in the sarcopenic and non-sarcopenic groups after WBV.

Few studies evaluated the acute effects of WBV stimulation on inflammatory biomarkers, especially TNF receptors, with only two studies being found (20,36). Thus, the mechanisms by which WBV alters sTNFr1 and sTNFr2 levels are not yet well established.

The current study had some limitations. The participants were older people from the general community of the city of Diamantina, MG, Brazil, and despite being sarcopenic, they were independent and had good physical function, hindering the extrapolation of the results to people with more severe sarcopenia and in other physical conditions. It was not possible to exclude some comorbidities common to older people that can be confounding factors, such as hypertension and diabetes; however, the distribution of these comorbidities was similar between groups.

In conclusion, the strength of the current study is that it investigated the acute response to WBV exercise of sTNFR levels in older people with sarcopenia. However, studies are needed to elucidate the mechanisms by which the addition of a WBV stimulus potentiates anti-inflammatory responses after exercising. This study demonstrated that a single acute session of WBV exercise can promote a higher modulation of sTNFr2 levels in sarcopenic people.
